# Association between soluble transferrin receptor and systolic hypertension in adults: National Health and Nutrition Examination Survey (2007–2010 and 2015–2018)

**DOI:** 10.3389/fcvm.2022.1029714

**Published:** 2022-11-04

**Authors:** Haoran Wang, Qianjin Qi, Shuaihua Song, Di Zhang, Li Feng

**Affiliations:** ^1^Shandong Provincial Hospital, Shandong University, Jinan, Shandong, China; ^2^Shandong Provincial Hospital Affiliated to Shandong First Medical University, Jinan, Shandong, China; ^3^Department of Clinical Nutrition, Shandong Provincial Hospital Affiliated to Shandong First Medical University, Jinan, Shandong, China

**Keywords:** hypertension, iron metabolism, soluble transferrin receptor, ferritin, National Health and Nutrition Examination Survey

## Abstract

**Background:**

Hypertension increases the global burden of disease and mortality. Iron metabolism is considered to be an important factor in hypertension. However, as an indicator of iron metabolism, little is known about the associations of soluble transferrin receptor (sTfR) with hypertension. We studied the relationship between sTfR and hypertension.

**Materials and methods:**

We studied 7,416 adults aged 20 years old or above from the National Health and Nutrition Examination Survey (NHANES), a nationally representative, cross-sectional, population-based study. Weighted logistic regression was used to examine the association between markers of iron metabolism and hypertension. The restricted cubic spline (RCS) was used to characterize the association between sTfR and blood pressure.

**Results:**

Weighted logistic regression showed that higher sTfR level was associated with higher odds of hypertension (OR = 1.05; 95% CI: 1.01–1.05; *p* = 0.001) after adjustment for all the potential confounding factors. Meanwhile, weighted logistic regression analyses indicated independent associations of high sTfR (*p* = 0.009) with systolic hypertension after adjusting for various different confounders. The result of restricted cubic splines showed a non-linear association between sTfR and systolic blood pressure among U.S. adults.

**Conclusion:**

Soluble transferrin receptor was found to be an independent factor in systolic hypertension. And, a non-linear relationship between sTfR and systolic blood pressure was discovered.

## Introduction

The prevalence and absolute burden of hypertension are rising globally, and hypertension is the leading cause of cardiovascular disease, including coronary heart disease, heart failure, stroke, myocardial infarction, atrial fibrillation, peripheral artery disease, chronic kidney disease (CKD), and cognitive impairment (1–3). According to the Global Burden of Disease Project, raised blood pressure was the greatest cause of global disease and mortality, accounting for 9.4 million deaths per year (4). Furthermore, a systematic analysis of the global burden of 87 risk factors in 204 countries and territories revealed that high systolic blood pressure was the leading Level 2 risk factor for attributable deaths, accounting for 108 million (95% UI) deaths [192 percent (169–213) of all deaths in 2019] (5). Many factors have been discovered to contribute to hypertension in recent research, including alcohol consumption, obesity, an unhealthy diet, and a lack of physical activity (6). However, there are still many unknown factors that influence the occurrence of hypertension which need further investigation.

Accumulating evidence has demonstrated that iron metabolism appears to play a significant role in the development of hypertension (7–9). However, previous studies that examined the association between markers of iron metabolism and hypertension have shown contradictory results. According to an Italian case-control study, elevated serum ferritin levels were prevalent in males with essential hypertension (10). Furthermore, a cross-sectional study in China found that hemoglobin and transferrin levels were linked to the occurrence of hypertension (11). However, a cohort study in France found that hemoglobin and ferritin levels were unrelated to blood pressure changes or accidental hypertension (12). Thus, more research is needed to explore the relationship between iron metabolism and hypertension.

In current study, soluble transferrin receptor (sTfR) and serum ferritin are commonly adapted to present the iron status. Compared with sTfR, serum ferritin was susceptible to other factors (e.g., inflammation) (13). sTfR was less affected by inflammation and reflected the degree of tissue iron supply (14–16). However, the associations of sTfR with hypertension-related traits have not been well evaluated. As a result, this study adapted the data from National Health and Nutrition Examination Survey (NHANES) to conduct a national population-based observational analysis to investigate the relationship between sTfRand hypertension.

## Research design and methods

This study adapted the data from National Health and Nutrition Examination Surveys (NHANES) 2007–2010 and NHANES 2015–2018. The NHANES survey used a stratified multistage random sample that was representative of the uninstitutionalized civilian population in U.S. The sample was drawn from a county, a neighborhood, a household, and the members of that household. To offer sufficient estimates of these racial groupings, it also included non-Hispanic blacks and Mexican Americans. Prior to participating, the subjects must sign a permission form and gain ethical clearance from the U.S. Department of Health and Human Services’ Human Subjects Committee (17).

**FIGURE 1:**
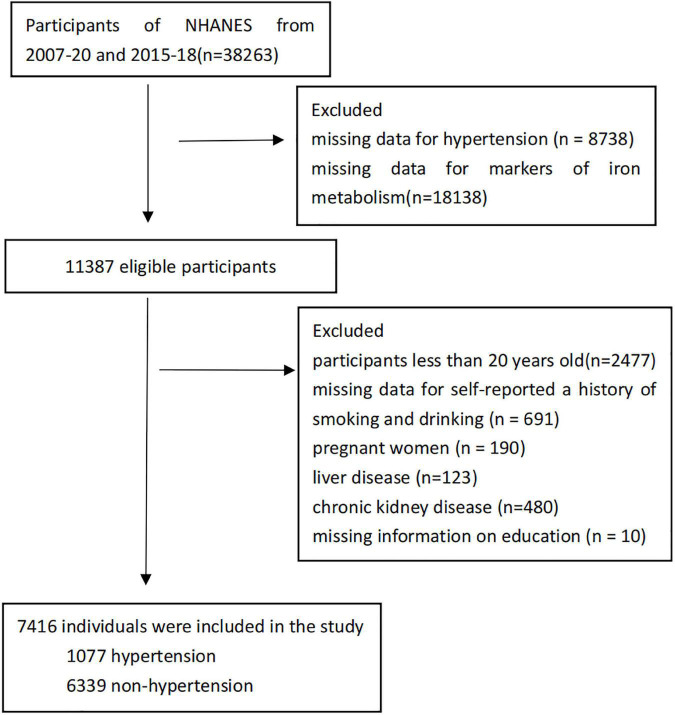
Flowchart of participant selection.

This study assessed 7,416 participants. The systolic blood pressure of at least 140 mmHg or a diastolic blood pressure of at least 90 mm Hg, or current use of an antihypertensive medication were classified as hypertension according to JNC-7 (18). Systolic hypertension was defined as having a systolic blood pressure of 140 mmHg or higher; diastolic hypertension was defined as having a diastolic blood pressure of 90 mmHg or higher (19). Exclusion criteria included missing data for hypertension (*n* = 8,738); missing data for markers of iron metabolism (*n* = 18,138); participants less than 20 years old (*n* = 2,477); missing data for self-reported a history of smoking and drinking (*n* = 691); pregnant women (*n* = 190); liver disease (*n* = 123, liver disease was defined as the subjects whose AST > 80 U/L or ALT > 80 U/L); chronic kidney disease (*n* = 480, Individuals with chronic kidney disease had an estimated glomerular filtration rate of less than 60 ml/min/1.73 m^2^) (20); missing information on education (*n* = 10). With the foregoing exclusion criteria in place, 7,416 individuals were included in the study, with 1,077 (14.5%) of them having hypertension (Figure 1).

### Main outcome measurements

Blood pressure was measured using a standardized methodology by trained examiners. According to American Heart Association and the Seventh Joint National Committee standards, sitting systolic and diastolic blood pressures (mm/Hg) were measured using a mercury sphygmomanometer after participants sat quietly for 5 min (18, 21). Blood pressure was taken three times, with a fourth reading taken if necessary. The means of systolic and diastolic blood pressure (SBP and DBP) were then computed (22).

In 2007–2010 and 2015–2018, serum ferritin concentration was measured by two methods. In 2007–2008, the measurements of serum ferritin concentration used the Roche Tina-quant serum ferritin immunoturbidimetric assay with the Hitachi 912 clinical analyzer (Roche Diagnostics, Basel, Switzerland). The method for measurement of serum ferritin concentrations was the Roche Elecsys 170 clinical analyzer in 2009–2010 and 2015–2018.

For NHANES 2007–2010 and 2015–2018, sTfR concentrations were measured at the National Center of Environmental Health, Center for Disease Control and Prevention. The Tina-quant sTfR test (Roche Diagnostics, Mannheim, Germany) was used to quantify sTfR concentrations with an automated homogeneous immunoturbidimetric assay. There was no change in laboratory procedure or site, but the test was run on three different chemical analyzers as follows (all made by Roche Diagnostics): Hitachi 912 (NHANES 2007–2008), Hitachi Mod P (NHANES 2009–2010 and 2015), and lastly, Roche c501 analyzers (NHANES 2016–2018). As a consequence of the same results for all three devices, no adjustments were necessary (23).

From 2003 to 2012, hemoglobin concentrations were tested by the mobile examination center using a Beckman Coulter MAXM hematology flow cytometer (Beckman Coulter, Fullerton, CA, USA), then switched to a Beckman Coulter Unicel DxH 800 Analyzer in 2013. To provide blood cell distribution for all participants, Beckman Coulter MAXM equipment was used to assess the complete blood count, including white blood cell count, at the NHANES Mobile Examination Center.

Serum iron was part of the standard biochemistry profile, which was measured at Collaborative Laboratory Services using a Beckman UniCel DxC800 Synchron for NHANES 2015–2016 and Beckman Synchron LX20 for NHANES 2007–2010, and at the University of Minnesota Advanced Research Diagnostics Laboratory using a Roche Cobas 6000 (c501 module) analyzer for NHANES 2017–2018.

To remove the difference of labs and assay techniques during data collection period. The NHANES group conducted crossover experiments to compare the data due to method changes amongst various assays, and adjustments were made accordingly based on the comparison before the data were released to the public (23–25).

### Covariate

The literature on hypertension risk factors was used to choose covariate (11, 12, 14). Age (years), gender (male/female), poverty income ratio (≤130%/>130%) (23), race/ethnicity (Mexican American, Other Hispanic, Non-Hispanic White, Non-Hispanic Black, Other Race–Including Multi-Racial), smoking status (never/former/current), drinking status (never/former/current), level of education (less than high school, high school or equivalent, college or above), and physical activity (active/inactive) were assessed using a questionnaire. According to the 2018 Physical Activity Guidelines Advisory Committee Scientific Report, participants were classed as inactive if they reported fewer than 10 min of moderate-to-vigorous physical activity per week (26). The body mass index (BMI) (kg/m^2^) was computed by dividing the weight in kilograms by the height in meters squared. Waist circumference (cm), triglycerides (mmol/L), total cholesterol (mmol/L), high-density lipoprotein (HDL) cholesterol (mmol/L), albumin (g/dL), total white blood cell count (SI), glycohemoglobin (%), and plasma fasting glucose (mmol/L) were also included as covariate. At the NHANES website, details regarding the measurement of the covariate were obtained (27).

### Statistical analyses

The data were processed according to the tutorials provided by NHANES; Including that the data were weighted according to the sample weight and multi period combination weight, as well as adjusted for the underestimation of variance due to the design scheme. For continuous variables, data were expressed as weighted averages with weighted standard deviations, and for categorical variables, data were expressed as weighted percentages. The hypertension and non-hypertensive groups were compared using the weighted chi-square test (categorical variables) and weighted Student’s *t*-tests (continuous variables). The levels of serum iron, serum ferritin, hemoglobin, and sTfR were categorized into quartiles. We utilized the lowest quartile as a reference and used weighted logistic regression models to calculate the odds ratio (OR) and 95 percent confidence interval (CI) for hypertension in each quartile. Simultaneously, *p*-trend analysis was performed in the weighted regression analyses by considering the quartiles as a continuous variable. Four models were developed: model 1 was adjusted for age and sex; model 2 was further adjusted for ethnicity, educational level, income, smoking, drinking, physical activity, BMI and waist circumference; model 3 was further adjusted for triglycerides, total cholesterol, HDL cholesterol, plasma fasting glucose and glycohemoglobin; and model 4 was further adjusted for white cell count and albumin. Collinearity diagnostics were carried out using the variance inflation factor (VIF). In order to further explore the relationship between iron metabolism markers and systolic and diastolic hypertension, weighted regression analysis was performed on iron metabolism markers and systolic and diastolic hypertension, respectively, and other covariates were adjusted. All the regressions above were statistically weighted (28).

Subgroup analyses were performed according to age, gender, menopause status, poverty income ratio, and physical activity to investigate the relationships between sTfR and systolic hypertension; and potential interactions were tested. Using exploratory data analysis techniques with R, we examined monotonic relationships between sTfR and systolic blood pressure in all participants, stratified by age. To further illustrate the correlation between sTfR and systolic blood pressure, we modeled sTfR against systolic blood pressure and also used a restricted cubic spline with five knots located at the 5th, 27.5th, 50th, 72.5th, and 95th percentiles to flexibly model the underlying relationship. All analyses were performed using R version 4.1.2, and a *p*-value < 0.05 was considered statistically significant.

## Results

High blood pressure was present in 1,077 of the 7,416 participants. Table 1 showed that the overall baseline characteristics of all individuals. Hypertensive participants were older, more likely to be smokers and drinkers, and had a lower level of education and physical activity. Hypertensive participants also had higher BMI, glycohemoglobin, fasting glucose, total cholesterol, triglyceride levels, white cell count, hemoglobin, ferritin, and sTfR levels. Furthermore, hypertensive participants had decreased HDL cholesterol and serum albumin. Participants who were non-Hispanic white, other Hispanic, and Mexican American had a decreased risk of hypertension.

**TABLE 1:** Characteristics of the study population [National Health and Nutrition Examination Survey (NHANES) 2007–2010 and NHANES 2015–2018].

Characteristics	Non-hypertension (*n* = 6,339)	Hypertension (*n* = 1,077)	*P*-value
Female (%)	74.1	56.9	<0.001
Physical activity (%)			<0.001
Active	76.7	68.9	
Inactive	23.3	31.1	
Smoking (%)			<0.001
Never	60.6	51.9	
Former	19.4	29.9	
Current	20.0	18.2	
Drinking (%)			<0.001
Never	9.3	8.6	
Former	13.9	21.5	
Current	76.8	69.9	
Poverty income ratio (%)			0.651
≤130%	20.2	19.3	
>130%	79.8	80.7	
Race-Hispanic origin (%)			<0.001
Mexican American	9.8	7.6	
Other Hispanic	6.7	6.3	
Non-Hispanic White	63.9	60.5	
Non-Hispanic Black	10.6	17.4	
Other Race–Including Multi-Racial	9.0	8.2	
Education (%)			<0.001
Less than high school	11.7	14.6	
High school or equivalent	22.8	28.5	
College or above	65.5	56.9	
Age (year)	40.12 ± 0.36	54.69 ± 0.62	<0.001
Body Mass Index (kg/m^2^)	29.05 ± 0.19	32.42 ± 0.29	<0.001
Waist circumference (cm)	96.89 ± 0.44	107.52 ± 0.70	<0.001
Glycohemoglobin (%)	5.48 ± 0.01	5.93 ± 0.05	<0.001
Glucose, plasma (mmol/L)	5.23 ± 0.02	5.98 ± 0.13	<0.001
Total cholesterol (mmol/L)	4.83 ± 0.03	5.13 ± 0.04	<0.001
HDL cholesterol (mmol/L)	1.43 ± 0.01	1.38 ± 0.02	0.004
Triglycerides (mmol/L)	1.46 ± 0.03	1.81 ± 0.04	<0.001
Total white blood cell count (SI)	7.38 ± 0.05	7.68 ± 0.11	0.010
Albumin (g/dL)	4.16 ± 0.01	4.07 ± 0.02	<0.001
Serum iron (ug/dL)	84.00 ± 1.03	85.67 ± 1.53	0.396
Ferritin (ug/L)	98.01 ± 2.59	146.13 ± 6.53	<0.001
Hemoglobin (g/dL)	13.80 ± 0.04	14.18 ± 0.08	<0.001
Transferrin receptor (mg/L)	3.30 ± 0.04	3.51 ± 0.08	0.009

Data are means ± SE (for continuous variables) or % (for categorical variables) unless otherwise indicated. All values presented are weighted to represent the U.S. civilian population, 2007–2010 and 2015–2018. *p*-value represents differences in means (SE) or proportions, using weighted Student’s *t*-tests (continuous variables) and the weighted chi-square test (categorical variables). HDL, high-density lipoprotein.

**FIGURE 2:**
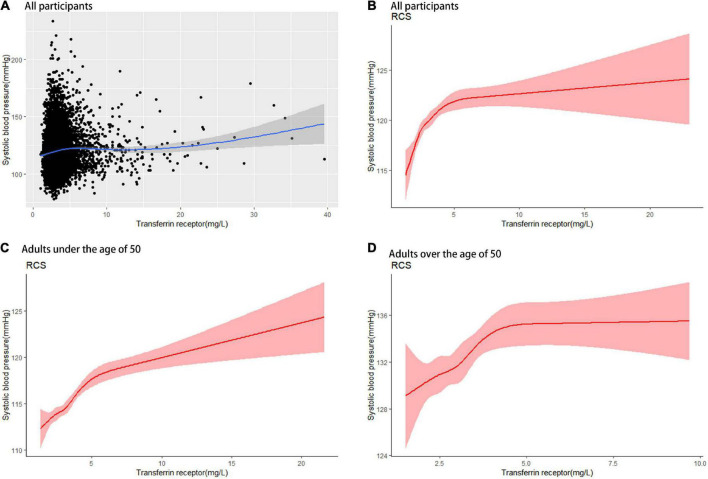
RCS of sTfR with systolic blood pressure. **(A)** sTfR concentrations with systolic blood pressure in all participants. **(B)** sTfR restricted cubic spline regression with 5 knots in all participants; shaded areas inside the dashed lines are 95% CIs. **(C)** sTfR restricted cubic spline regression with 5 knots in adults under the age of 50; shaded areas inside the dashed lines are 95% CIs. **(D)** sTfR restricted cubic spline regression with 5 knots in adults over the age of 50; shaded areas inside the dashed lines are 95% CIs. sTfR, soluble transferrin receptor; RCS, restricted cubic spline.

As shown in Table 2, higher sTfR levels were associated with higher odds of hypertension (OR = 1.05; 95% CI: 1.01–1.09; *p* = 0.001) after adjustment for all the potential confounding factors, including age, sex, ethnicity, educational level, income, smoking, drinking, physical activity, BMI, waist circumference, triglycerides, total cholesterol, HDL cholesterol, fasting glucose, glycohemoglobin, white cell count, and albumin. The model for evaluating the trend of this association was also statistically significant (*p* < 0.05). Hemoglobin, ferritin and serum iron had a non-significant effect on the risk of hypertension. Additionally, weighted logistic regression analysis was performed to assess the association between markers of iron metabolism and systolic/diastolic hypertension. As shown in Table 3, sTfR was positively associated with systolic hypertension (OR = 1.03; 95% CI: 1.01–1.06; *p* = 0.009) after adjustment for the variables. The model for evaluating the trend of this association was also statistically significant (*p* < 0.05). However, there were no significant relationships between sTfR level and diastolic hypertension, as shown in Table 4 (OR = 1.02; 95% CI: 0.99–1.05; *p* = 0.148). Linear regression was performed to evaluate the association of sTfR with SBP and DBP, respectively. The result showed that sTfR was statistically significantly correlated with SBP (Supplementary Table 1), while sTfR was not statistically significantly correlated with DBP (Supplementary Table 2). The empirical distribution plots indicated clear non-linear trends at certain concentrations of sTfR (Figure 2A). Restricted cubic spline model was to further explore the non-linear association between sTfR and systolic blood pressure. First, there was a non-linear relationship between sTfR and systolic blood pressure in all participants (Figure 2B). In adults under the age of 50, there was a non-linear association between sTfR and systolic blood pressure (Figure 2C), and the test for non-linearity was significant (*p* < 0.05). To the left of the inflection point, the influence was substantially enhanced; to the right, the effect was flat. In adults over 50, the association between sTfR and systolic blood pressure was likewise non-linear (Figure 2D), with the non-linear test being highly significant (*p* < 0.05). Similarly, the influence was greatly boosted on the left side of the inflection point, while the effect was flat on the right.

**TABLE 2:** Association between markers of iron metabolism (as quartile) and hypertension in National Health and Nutrition Examination Survey (NHANES) 2007–2010 and NHANES 2015–2018.

Iron indices	Model	Quartile 1 (reference)	Quartile 2	Quartile 3	Quartile 4	*P*-trend
Serum iron	*N* (*n*)	1,941 (246)	1,797 (282)	1,840 (307)	1,838 (242)	
Median (Q1, Q3)		44 (32, 52)	69 (64, 74)	90 (84, 97)	124 (113, 144)	
	1	1	1.00 (0.98–1.01)	1.00 (0.98–1.03)	1.00 (0.98–1.04)	0.766
	2	1	1.01 (0.99–1.02)	1.02 (0.99–1.05)	1.03 (1.01–1.07)*	0.078
	3	1	1.00 (0.98–1.02)	1.02 (0.99–1.04)	1.03 (1.00–1.06)	0.139
	4	1	1.00 (0.98–1.02)	1.02 (0.99–1.05)	1.03 (1.00–1.06)	0.146
Ferritin	*N* (*n*)	1,856 (169)	1,853 (177)	1,862 (291)	1,845 (440)	
Median (Q1, Q3)		17.3 (10.0, 24.9)	47.0 (39.0, 57.7)	95.0 (80.0, 113.0)	213.0 (166.0, 302.0)	
	1	1	1.00 (0.98–1.03)	1.02 (0.99–1.06)	1.06 (1.01–1.11)*	0.127
	2	1	1.00 (0.97–1.02)	1.01 (0.98–1.04)	1.04 (0.99–1.08)	0.320
	3	1	0.99 (0.97–1.02)	1.01 (0.98–1.04)	1.03 (0.99–1.08)	0.375
	4	1	0.99 (0.97–1.02)	1.01 (0.97–1.04)	1.03 (0.98–1.08)	0.385
Hemoglobin	*N* (*n*)	1,865 (349)	2,042 (219)	1,773 (244)	1,736 (349)	
Median (Q1, Q3)		12.3 (11.6, 12.7)	13.4 (13.2, 13.6)	14.1 (13.9, 14.4)	15.4 (15.0, 15.9)	
	1	1	0.99 (0.97–1.02)	1.00 (0.97–1.02)	1.02 (0.97–1.07)	0.028
	2	1	0.99 (0.97–1.02)	1.00 (0.97–1.02)	1.01 (0.97–1.06)	0.092
	3	1	0.99 (0.96–1.02)	0.99 (0.97–1.02)	1.01 (0.96–1.05)	0.141
	4	1	0.99 (0.97–1.02)	0.99 (0.97–1.02)	1.00 (0.96–1.05)	0.200
Soluble Transferrin Receptor	*N* (*n*)	1,865 (170)	1,861 (250)	1,844 (295)	1,846 (362)	
Median (Q1, Q3)		2.05 (1.90, 2.26)	2.65 (2.51, 2.80)	3.24 (3.09, 3.43)	5.52 (4.00, 5.70)	
	1	1	1.01 (0.98–1.04)	1.06 (1.03–1.08)*	1.07 (1.04–1.11)*	<0.001
	2	1	1.00 (0.98–1.03)	1.05 (1.02–1.07)*	1.05 (1.02–1.09)*	<0.001
	3	1	1.01 (0.98–1.03)	1.04 (1.02–1.07)*	1.05 (1.02–1.09)*	<0.001
	4	1	1.01 (0.98–1.03)	1.04 (1.02–1.07)*	1.05 (1.01–1.09)*	0.001

Model 1: Adjusted for age and sex.

Model 2: Further adjusted for ethnicity, educational level, income, smoking, drinking, physical activity, BMI, and waist circumference.

Model 3: Further adjusted for triglycerides, total cholesterol, HDL cholesterol, fasting glucose, and glycohemoglobin.

Model 4: Further adjusted for white cell count and albumin. Data are expressed as OR (95% CI).

*p < 0.05.

**TABLE 3:** Association between markers of iron metabolism (as quartile) and systolic hypertension in National Health and Nutrition Examination Survey (NHANES) 2007–2010 and NHANES 2015–2018.

Iron indices	Model	Quartile 1 (reference)	Quartile 2	Quartile 3	Quartile 4	*P*-trend
Serum iron	*N* (*n*)	1,941 (196)	1,797 (237)	1,840 (247)	1,838 (200)	
Median (Q1, Q3)		44 (32, 52)	69 (64, 74)	90 (84, 97)	124 (113, 144)	
	1	1	1.00 (0.98–1.02)	1.01 (0.98–1.04)	1.00 (0.98–1.03)	0.838
	2	1	1.01 (0.99–1.03)	1.02 (0.99–1.05)	1.02 (0.99–1.05)	0.080
	3	1	1.01 (0.99–1.03)	1.02 (0.99–1.05)	1.02 (0.99–1.05)	0.132
	4	1	1.01 (0.99–1.03)	1.02 (0.99–1.05)	1.02 (0.99–1.05)	0.135
Ferritin	*N* (*n*)	1,856 (132)	1,853 (151)	1,862 (232)	1,845 (365)	
Median (Q1, Q3)		17.3 (10.0, 24.9)	47.0 (39.0, 57.7)	95.0 (80.0, 113.0)	213.0 (166.0, 302.0)	
	1	1	1.00 (0.99–1.02)	1.01 (0.98–1.03)	1.03 (1.00–1.07)	0.425
	2	1	1.00 (0.98–1.02)	1.00 (0.97–1.02)	1.02 (0.99–1.06)	0.804
	3	1	1.00 (0.98–1.02)	1.00 (0.97–1.02)	1.02 (0.99–1.06)	0.831
	4	1	1.00 (0.98–1.02)	1.00 (0.97–1.02)	1.02 (0.98–1.05)	0.851
Hemoglobin	*N* (*n*)	1,865 (227)	2,042 (183)	1,773 (192)	1,736 (278)	
Median (Q1, Q3)		12.3 (11.6, 12.7)	13.4 (13.2, 13.6)	14.1 (13.9, 14.4)	15.4 (15.0, 15.9)	
	1	1	0.99 (0.97–1.01)	0.99 (0.97–1.01)	1.01 (0.97–1.04)	0.242
	2	1	0.99 (0.97–1.01)	0.99 (0.97–1.01)	1.00 (0.96–1.03)	0.530
	3	1	0.99 (0.97–1.02)	0.99 (0.97–1.01)	1.00 (0.96–1.04)	0.521
	4	1	0.99 (0.97–1.02)	0.99 (0.97–1.02)	1.00 (0.96–1.05)	0.676
Soluble Transferrin Receptor	*N* (*n*)	1,865 (144)	1,861 (209)	1,844 (240)	1,846 (287)	
Median (Q1, Q3)		2.05 (1.90, 2.26)	2.65 (2.51, 2.80)	3.24 (3.09, 3.43)	5.52 (4.00, 5.70)	
	1	1	1.00 (0.98–1.03)	1.04 (1.02–1.06)*	1.05 (1.02–1.07)*	<0.001
	2	1	1.00 (0.98–1.03)	1.04 (1.01–1.06)*	1.03 (1.01–1.06)*	0.003
	3	1	1.00 (0.98–1.03)	1.03 (1.01–1.06)*	1.03 (1.01–1.06)*	0.005
	4	1	1.00 (0.98–1.03)	1.03 (1.01–1.06)*	1.03 (1.01–1.06)*	0.009

Model 1: Adjusted for age and sex.

Model 2: Further adjusted for ethnicity, educational level, income, smoking, drinking, physical activity, BMI, and waist circumference.

Model 3: Further adjusted for triglycerides, total cholesterol, HDL cholesterol, fasting glucose, and glycohemoglobin.

Model 4: Further adjusted for white cell count and albumin. Data are expressed as OR (95% CI).

*p < 0.05.

**TABLE 4:** Association between markers of iron metabolism (as quartile) and diastolic hypertension in National Health and Nutrition Examination Survey (NHANES) 2007–2010 and NHANES 2015–2018.

Iron indices	Model	Quartile 1 (reference)	Quartile 2	Quartile 3	Quartile 4	*P*-trend
Serum iron	*N* (*n*)	1,941 (103)	1,797 (109)	1,840 (109)	1,838 (100)	
Median (Q1, Q3)		44 (32, 52)	69 (64, 74)	90 (84, 97)	124 (113, 144)	
	1	1	1.00 (0.98–1.02)	1.00 (0.98–1.01)	1.01 (0.99–1.04)	0.969
	2	1	1.01 (0.98–1.03)	1.01 (0.99–1.02)	1.03 (1.00–1.05)	0.189
	3	1	1.00 (0.98–1.03)	1.00 (0.99–1.02)	1.02 (1.00–1.05)	0.223
	4	1	1.01 (0.98–1.03)	1.00 (0.99–1.02)	1.02 (0.99–1.05)	0.215
Ferritin	*N* (*n*)	1,856 (82)	1,853 (60)	1,862 (109)	1,845 (171)	
Median (Q1, Q3)		17.3 (10.0, 24.9)	47.0 (39.0, 57.7)	95.0 (80.0, 113.0)	213.0 (166.0, 302.0)	
	1	1	0.99 (0.97–1.00)	1.01 (0.98–1.04)	1.01 (0.98–1.04)	0.169
	2	1	0.98 (0.96–1.00)*	1.00 (0.98–1.03)	1.00 (0.97–1.03)	0.302
	3	1	0.98 (0.96–0.99)*	1.00 (0.97–1.03)	1.00 (0.97–1.02)	0.389
	4	1	0.98 (0.96–0.99)*	1.00 (0.97–1.03)	1.00 (0.97–1.02)	0.388
Hemoglobin	*N* (*n*)	1,865 (92)	2,042 (63)	1,773 (90)	1,736 (176)	
Median (Q1, Q3)		12.3 (11.6, 12.7)	13.4 (13.2, 13.6)	14.1 (13.9, 14.4)	15.4 (15.0, 15.9)	
	1	1	1.00 (0.98–1.01)	1.00 (0.98–1.03)	1.03 (1.01–1.06)*	0.007
	2	1	0.99 (0.98–1.01)	1.00 (0.98–1.02)	1.03 (1.00–1.05)	0.027
	3	1	0.99 (0.97–1.01)	1.00 (0.98–1.02)	1.02 (0.99–1.05)	0.070
	4	1	0.99 (0.97–1.01)	1.00 (0.98–1.02)	1.02 (1.00–1.05)	0.040
Soluble Transferrin Receptor	*N* (*n*)	1,865 (63)	1,861 (99)	1,844 (111)	1,846 (148)	
Median (Q1, Q3)		2.05 (1.90, 2.26)	2.65 (2.51, 2.80)	3.24 (3.09, 3.43)	5.52 (4.00, 5.70)	
	1	1	1.00 (0.98–1.02)	1.03 (1.01–1.05)*	1.04 (1.01–1.07)*	<0.001
	2	1	1.00 (0.98–1.02)	1.02 (1.00–1.05)	1.03 (1.00–1.06)	0.001
	3	1	1.00 (0.98–1.02)	1.02 (1.00–1.04)	1.02 (0.99–1.05)	0.004
	4	1	1.00 (0.98–1.02)	1.02 (1.00–1.04)	1.02 (0.99–1.05)	0.003

Model 1: Adjusted for age and sex.

Model 2: Further adjusted for ethnicity, educational level, income, smoking, drinking, physical activity, BMI, and waist circumference.

Model 3: Further adjusted for triglycerides, total cholesterol, HDL cholesterol, fasting glucose, and glycohemoglobin.

Model 4: Further adjusted for white cell count and albumin. Data are expressed as OR (95% CI).

*p < 0.05.

Subgroup analyses were performed to further assess the robustness of the association between sTfR and systolic hypertension. All analyses were adjusted for age, gender, ethnicity, poverty income ratio, smoking status, drinking status, level of education, physical activity, BMI, waist circumference, triglycerides, total cholesterol, HDL, albumin, total white blood cell count, glycohemoglobin, plasma fasting glucose, and menopause status, except for the variable that was stratified. In female participants, those with the highest quartile of sTfR levels had a significantly higher risk of systolic hypertension when compared to the lowest group (Supplementary Table 3). However, this association was not found in male participants (Supplementary Table 4). The associations of sTfR and systolic hypertension were significant in participants with a lower poverty income ratio (Supplementary Figure 1). Potential interactions between sTfR and the stratification factors were also tested. No interaction between sTfR and other covariates was found (Supplementary Figure 1).

## Discussion

This nationwide population-based study indicated that transferrin, serum iron, and hemoglobin levels had no significant effect on the risk of hypertension. sTfR was positively associated with systolic hypertension but not diastolic hypertension. Furthermore, this study found a non-linear association between sTfR and systolic blood pressure. Our findings showed that sTfR was a significant independent risk factor for the hypertension in the American population.

This study showed that hemoglobin and serum iron levels were not associated with hypertension. However, according to a Dutch study, hemoglobin had a substantial positive relationship with blood pressure (29). Samuels et al. (30) reported that serum iron was increased in 30 pregnant women with pregnancy-induced hypertension. Compared with the previous two studies, the study population was more representative and the sample size was larger. Previous research had confirmed the lack of a link between ferritin and hypertension. A Chinese cohort research found that serum ferritin content had no effect on the occurrence of hypertension (11). And, a cohort study in France suggested that ferritin concentrations were not associated with changes in blood pressure or incidental hypertension (12). However, previous studies in Korea suggested that ferritin was positively associated with the prevalence of hypertension (31, 32). This variation in results could be attributed to a variety of reasons, including the study population’s and study area’s heterogeneity, as well as study methods.

The cross-sectional study from the U.S. showed sTfR was the biomarker associated with the risk of uncontrolled hypertension compared to controlled hypertension (33). Recently, some studies showed that elevated sTfR was related to several cardiovascular diseases, such as chronic left heart failure and coronary heart disease (34–36). Because serum ferritin concentrations could be influenced by inflammation, sTfR, which was less influenced by inflammation, might have aided in the interpretation of the relationship between iron metabolism and hypertension (37, 38). Zhu et al. (11) reported that the sTfR level was positively correlated with both SBP and DBP, but the study did not detect any association of the sTfR level with incident hypertension in the Chinese population. The study was required to confirm the association between the full range of serum ferritin, sTfR, serum iron, and hemoglobin concentrations with hypertension in different ethnic groups.

Our study showed that sTfR was an independent risk factor for systolic hypertension but not diastolic hypertension, and it also presented a curvilinear relationship between sTfR and systolic blood pressure. A cross-sectional study in Spain showed that sTfR was correlated with systolic blood pressure, but not diastolic blood pressure (39). Even after adjusting for various different confounders such as urine sodium and potassium, Tzoulaki et al. observed that non-heme iron and total iron intakes were connected with reduced SBP in a cross-sectional dietary study (40). The sample size of the cross-sectional study in Spain was too small (39), and dietary assessments of the cross-sectional dietary study depended on reporting by participants (40). Our study did not have the above two shortcomings. Furthermore, exploratory analysis found curvilinear relationships between sTfR concentration and systolic blood pressure (Figure 2A). Then, we identified a relationship between sTfR and systolic blood pressure using restricted cubic spline analysis in the combined NHANES 2007–2010 and 2015–2018 data (Figure 2B). For modeling the non-linear relationship between a continuous variable (e.g., sTfR) and an outcome (e.g., systolic blood pressure), restricted cubic spline analysis is beneficial. Our findings suggested that a non-linear association between sTfR and systolic blood pressure was observed in both the elderly and the young (Figures 2C,D). As a marker of iron metabolism, sTfR was an independent risk factor of systolic hypertension. In the future, sTfR might be regulated to treat systolic hypertension.

Although the mechanism underlying this impact is unknown, it lends credence to the theory that iron availability influences hemodynamic control. Lindberg et al. (41) hypothesized that iron might impact blood pressure in the systemic or pulmonary circulation *via* nitric oxide (NO), a regulatory chemical that promoted vascular smooth-muscle cell relaxation. NO synthase was responsible for the production of NO, and iron was a necessary component (7). As a result, iron availability might influence NO synthesis. Iron deficiency had been shown to impair NO-synthase activity in rats (42), and we hypothesized that low iron availability (associated with increased sTfR) might result in higher blood pressure, which was mediated by a lack of NO generation. When sTfR increased to about 5 mg/L, the effect of iron deficiency on systolic blood pressure decreased significantly. To approve this hypothesis, further mechanism study is still needed. Our study provided a new direction for the effective treatment of systolic hypertension.

Our research has some significant advantages. Firstly, the data in our study was from a large, representative sample of the general population of the United States. Secondly, restricted cubic spline was used to explore the non-linear relationship between sTfR and systolic blood pressure. There are several potential imitations of this study as well. First, the cross-sectional data analysis of the connections between indicators of iron status and hypertension hindered us from drawing a causal conclusion regarding the association. Second, even though confounder amounts were adjusted, we can’t rule out the possibility that unmeasured or residual confounders influenced the associations reported in our study. Third, in different cycles, different testing instruments were used for the detection of the same indicator, and the results might be different. For example, in 2015–2018, high-sensitivity CRP (HS-CRP) was adapted to replace CRP measurements (2007–2010). Therefore, this indicator was not adjusted.

## Conclusion

In conclusion, this study indicated that sTfR was an independent factor in systolic hypertension. And, we found that the relationship of sTfR with systolic blood pressure was non-linear among U.S. adults. Our results might provide a new point of view on the roles of markers of iron status in hypertension related metabolic dysfunctions.

## Data availability statement

The datasets presented in this study can be found in online repositories. The names of the repository/repositories and accession number(s) can be found below: https://www.cdc.gov/nchs/nhanes/index.htm.

## Ethics statement

The NHANES implementation protocol was approved and conducted by Centers for Disease Control and Prevention (CDC) National Center for Health Statistics (NCHS). The patients/participants provided their written informed consent to participate in this study.

## Author contributions

HW wrote the first draft of the manuscript. All authors commented on previous versions of the manuscript, contributed to the study conception and design, performed material preparation, data collection, and data analysis, read, and approved the final manuscript.
