# Supplementary material for: Association between soluble transferrin receptor and systolic hypertension in adults: National Health and Nutrition Examination Survey (2007–2010 and 2015–2018)
Source: Front Cardiovasc Med. 2022 Nov 4;9:1029714. doi: 10.3389/fcvm.2022.1029714 (PMC9671951; doi:10.3389/fcvm.2022.1029714)
Supplement: Supplementary file 1 [file Data_Sheet_1.PDF]

Supplementary Material

1 Supplementary Tables

Supplementary Table S1 Associations of soluble transferrin receptor with systolic blood pressure in adults at baseline.

	Coef (95% CI)	p-value
Model 1	0.668(0.406, 0.929)	<0.001
Model 2	0.410(0.182, 0.638)	<0.001
Model 3	0.417(0.188, 0.647)	<0.001
Model 4	0.430(0.194, 0.666)	<0.001

Model 1: Adjusted for age and sex; Model 2: Further adjusted for ethnicity, educational level, income, smoking, drinking, physical activity, BMI and waist circumference; Model 3: Further adjusted for triglycerides, total cholesterol, HDL cholesterol, fasting glucose and glycohemoglobin; Model 4: Further adjusted for white cell count and albumin; Data are expressed as Coef (95% CI).

Supplementary Table S2 Associations of soluble transferrin receptor with diastolic blood pressure in adults at baseline.

	Coef (95% CI)	*p*-value
Model 1	0.093(−0.112, 0.299)	0.368
Model 2	0.039(−0.237, 0.159)	0.694
Model 3	−0.002(−0.198, 0.194)	0.981
Model 4	0.003(−0.201, 0.207)	0.975

Model 1: Adjusted for age and sex; Model 2: Further adjusted for ethnicity, educational level, income, smoking, drinking, physical activity, BMI and waist circumference; Model 3: Further adjusted for triglycerides, total cholesterol, HDL cholesterol, fasting glucose and glycohemoglobin; Model 4: Further adjusted for white cell count and albumin; Data are expressed as Coef (95% CI).

Supplementary Table S3 Association between soluble transferrin receptor (as quartile) and systolic hypertension in female.

	Model N(n)	Quartile 1 (reference) 1224(77)	Quartile 2 1258(112)	Quartile 3 1357(148)	Quartile 4 1615(175)	*P* trend
Median (Q1, Q3)		2.10(1.90, 2.30)	2.70(2.58, 2.82)	3.30(3.11, 3.50)	4.70(4.10, 6.10)	
	1	1	0.99(0.97−1.02)	1.03(1.01−1.05) *	1.04(1.01−1.07) *	<0.001
	2	1	0.99(0.96−1.02)	1.02(0.99−1.04)	1.03(1.01−1.05) *	0.015
	3	1	0.99(0.96−1.02)	1.02(0.99−1.04)	1.03(1.01−1.05) *	0.025
	4	1	0.99(0.96−1.02)	1.02(0.99−1.04)	1.03(1.01−1.05) *	0.035

Model 1: Adjusted for age; Model 2: Further adjusted for ethnicity, educational level, income, smoking, drinking, physical activity, BMI and waist circumference; Model 3: Further adjusted for triglycerides, total cholesterol, HDL cholesterol, fasting glucose and glycohemoglobin; Model 4: Further adjusted for white cell count, albumin and menopause status; Data are expressed as OR (95% CI). * $P < 0.05$.

Supplementary Table S4 Association between soluble transferrin receptor (as quartile) and systolic hypertension in male.

	Model N(n)	Quartile 1 (reference) 427(69)	Quartile 2 478(88)	Quartile 3 502(85)	Quartile 4 555(126)	*P* trend
Median (Q1, Q3)		2.11(1.93, 2.26)	2.59(2.46, 2.70)	3.07(2.93, 3.24)	4.08(3.71, 4.81)	
	1	1	0.98(0.96−1.02)	1.03(1.01−1.04) *	1.04(1.01−1.06) *	0.083
	2	1	0.98(0.94−1.01)	1.01(0.99−1.03)	1.03(0.99−1.05)	0.087

3	1	0.98(0.94–1.01)	1.01(0.99–1.03)	1.03(0.99–1.05)	0.347
4	1	0.98(0.94–1.01)	1.01(0.99–1.03)	1.03(0.99–1.05)	0.432

Model 1: Adjusted for age; Model 2: Further adjusted for ethnicity, educational level, income, smoking, drinking, physical activity, BMI and waist circumference; Model 3: Further adjusted for triglycerides, total cholesterol, HDL cholesterol, fasting glucose and glycohemoglobin; Model 4: Further adjusted for white cell count and albumin; Data are expressed as OR (95% CI). * *P* < 0.05.

2 Supplementary Figures

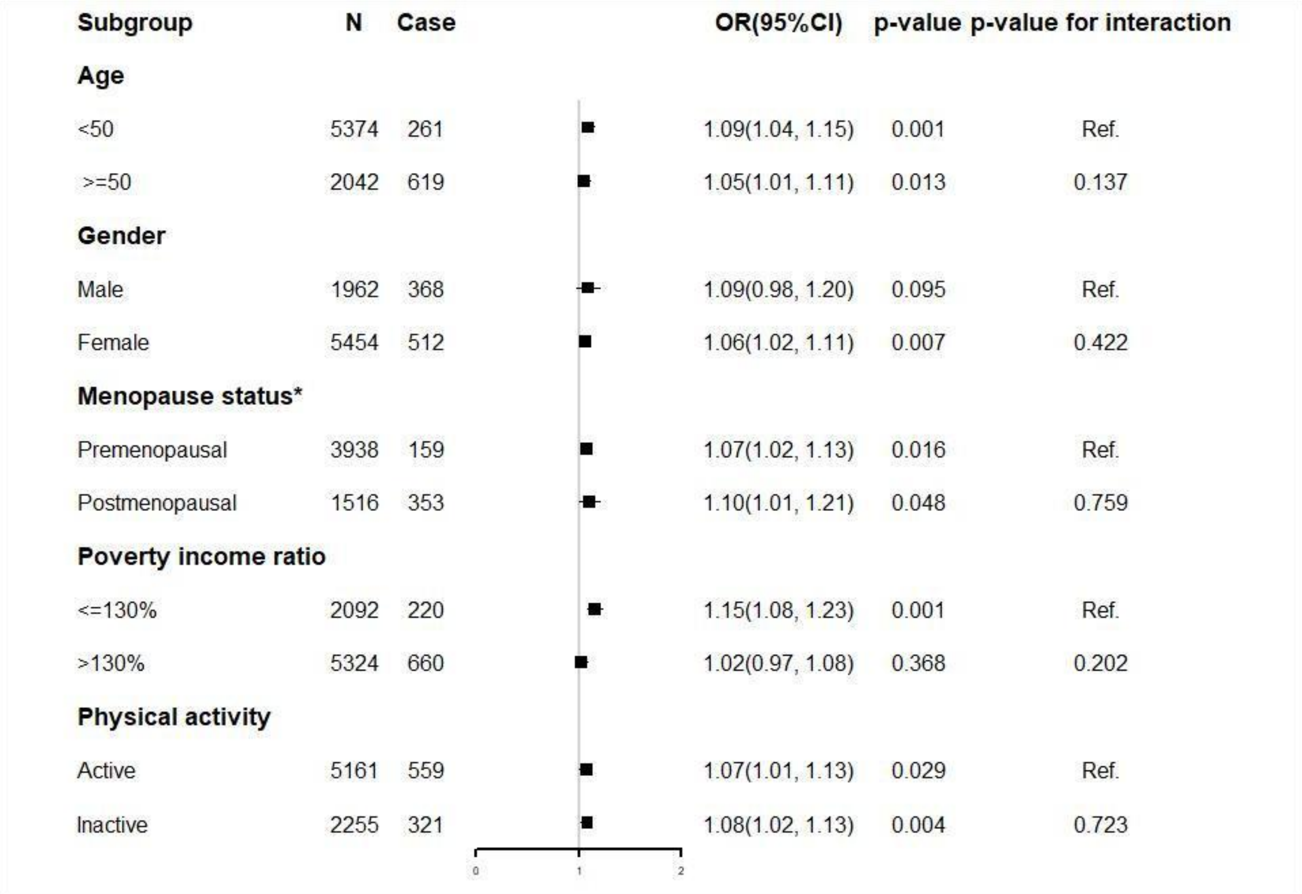

Supplementary Figure S1. Subgroup analysis Odds ratio of sTfR for systolic hypertension, adjusted for age, gender, ethnicity, poverty income ratio, smoking status, drinking status, level of education, physical activity, BMI, waist circumference, triglycerides, total cholesterol, HDL, albumin, total white blood cell count, glycohemoglobin, plasma fasting glucose and menopause status. Abbreviations: OR, odds ratio; CI, confidence interval; sTfR, soluble transferrin receptor. *all female participants.
